# Long term outcomes of hyperbaric oxygen therapy in post covid condition: longitudinal follow-up of a randomized controlled trial

**DOI:** 10.1038/s41598-024-53091-3

**Published:** 2024-02-15

**Authors:** Amir Hadanny, Shani Zilberman-Itskovich, Merav Catalogna, Karin Elman-Shina, Erez Lang, Shachar Finci, Nir Polak, Ran Shorer, Yoav Parag, Shai Efrati

**Affiliations:** 1 Sagol Center for Hyperbaric Medicine and Research, Shamir (Assaf Harofeh) Medical Center, Zerifin, Israel; 2 School of Medicine, Tel-Aviv University, Tel-Aviv, Israel; 3 Sagol School of Neuroscience, Tel-Aviv University, Tel-Aviv, Israel

**Keywords:** Neurological disorders, Infectious diseases

## Abstract

In our previous randomized controlled trial, we documented significant improvements in cognitive, psychiatric, fatigue, sleep, and pain symptoms among long Coronavirus disease 2019 (COVID) patients who underwent hyperbaric oxygen therapy (HBOT). The primary objective of the present study was to evaluate the enduring 1 year long term effects of HBOT on long COVID syndrome. This longitudinal long-term follow-up included 31 patients with reported post COVID-19 cognitive symptoms, who underwent 40 daily sessions of HBOT. Participants were recruited more than one year (486 ± 73) after completion of the last HBOT session. Quality of life, assessed using the short form-36 (SF-36) questionnaire revealed, that the long-term results exhibited a similar magnitude of improvement as the short-term outcomes following HBOT across most domains. Regarding sleep quality, improvements were observed in global score and across five sleep domains with effect sizes of moderate magnitude during the short-term evaluation, and these improvements persisted in the long-term assessment (effect size (ES1) = 0.47–0.79). In the realm of neuropsychiatric symptoms, as evaluated by the brief symptom inventory-18 (BSI-18), the short-term assessment following HBOT demonstrated a large effect size, and this effect persisted at the long-term evaluation. Both pain severity (ES1 = 0.69) and pain interference (ES1 = 0.83), had significant improvements during the short-term assessment post HBOT, which persisted at long term. The results indicate HBOT can improve the quality of life, quality of sleep, psychiatric and pain symptoms of patients suffering from long COVID. The clinical improvements gained by HBOT are persistent even 1 year after the last HBOT session.

## Introduction

Coronavirus disease 2019 (Covid-19) represents a current pandemic disease caused by a novel severe acute respiratory syndrome coronavirus type 2 (SARS-CoV-2) leading to formidable global effects. Long COVID, also known as post covid-19 condition, encompasses a range of symptoms that persist for weeks or months after the acute phase of infection with the severe acute respiratory syndrome coronavirus 2 (SARS-CoV-2). The occurrence of long COVID spans a wide spectrum, afflicting 10–30% of non-hospitalized cases, 50–70% of those who required hospitalization, and 10–12% of vaccinated cases^1–3^. The suggested pathogenesis of long COVID involves a multitude of mechanisms including immune system dysregulation, exaggerated inflammatory response, hyperviscosity and microvascular damage, with more complex consequences in organs with high oxygen demand such as the central nervous system and the heart^4^. Numerous studies have explored the description, occurrence, and long-term consequences of the syndrome. However, a limited number of these studies have delved into interventions designed to ameliorate the core pathology and enhance clinical symptoms. Among these, the most promising treatment intervention, validated in well-controlled randomized controlled trials, has demonstrated the ability to induce notable neuroplasticity and substantially enhance clinical outcomes, involves the utilization of a novel hyperbaric oxygen therapy (HBOT) protocol^5,6^. The clinical benefits of HBOT were assessed within a timeframe of 1–3 weeks after the conclusion of the last HBOT sessions, while investigations into its long-term consequences have yet to be undertaken^5^.

The existing treatment options for long COVID are primarily informed by small-scale pilot studies within the context of long COVID or have drawn from successful approaches in managing other conditions^7^, such as the use of β-blockers for addressing postural orthostatic tachycardia syndrome (POTS)^7^, low-dose naltrexone for mitigating neuroinflammation^8^, intravenous immunoglobulin to address immune dysfunction^9^, tailored dietary approaches, and cognitive-behavioral therapy (CBT). However, the majority of these possibilities necessitate rigorous clinical testing, and expedited clinical trials are imperative^10^.

In recent years, a mounting body of evidence has emerged concerning the neuroplasticity-inducing effects of the new protocols of hyperbaric oxygen therapy (HBOT)^11–18^. HBOT entails the application of elevated atmospheric pressure in combination with increased oxygen levels, which enhances the diffusion of oxygen to poorly perfused tissues. The new protocol of HBOT, using the hyperoxic-hypoxic paradox (HHP), is one of the first therapeutic intervention already in clinical use today for the specific goal of inducing the regeneration of damaged brain tissue^11,12,17^. HHP is a newly suggested paradigm aiming to enhance the endogenous repair mechanisms, while providing an optimal microenvironment^11^. These effects include stem cells stimulation, migration and differentiation, mitochondrial proliferation/biogenesis, mitochondrial transfer and angiogenesis^11,12,17^. In our randomized controlled trial, we reported that HBOT can improve both cognitive, psychiatric, fatigue, sleep and pain symptoms in patients of long COVID^5^. However, one of the major limitations was that results were measured 1–3 weeks after the last HBOT sessions.

The aim of the current study was to evaluate the long term effects of HBOT on patients suffering from post-COVID-19 who participated in the randomized controlled trial.

## Results

### Patient characteristics

Patients’ baseline characteristics demographics, and high-risk comorbidities, are detailed in Table 1. No statistically significant differences in baseline characteristics were observed between the original study’s HBOT group and the current cohort with long term evaluation.

**Table 1:** Baseline characteristics.

	HBOT arm	Long term HBOT	P
N	37	31	
Age (Y)	48.4 ± 10.6	46.7 ± 11.5	0.532
Males	18 (48.6)	14 (45.2)	0.774
Female	19 (51.4)	17 (54.8)	0.774
BMI (kg/m^2^)	26.9 ± 5.1	26.8 ± 5.0	0.944
Years of education	14.6 ± 2.7	14.8 ± 2.5	0.845
Marital status			0.955
Single	5 (13.5)	5 (16.1)	
Married	27 (73.0)	23 (74.2)	
Divorced	3 (8.1)	2 (6.5)	
Widowed	2 (5.4)	1 (3.2)	
Number of children	2.5 ± 1.4	2.2 ± 1.5	0.422
Employment status	33 (89.2)	27 (87.1)	1.000 (F)
Time from infection (days)	159.1 ± 71.3	158.3 ± 76.1	0.989
MoCA—cognitive assessment	25.4 ± 3.6	25.5 ± 3.6	0.843
Hospitalized*	4 (10.8)	3 (9.7)	1.000 (F)
High risk conditions			
BMI^†^ > 30	11 (29.7)	9 (29.0)	1.000 (F)
Age > 60 Y	4 (10.8)	2 (6.5)	0.681 (F)
Cancer	0 (0.0)	0 (0.0)	1.000 (F)
Diabetes mellitus	1 (2.7)	1 (3.2)	1.000 (F)
Hypertension	4 (10.8)	3 (9.7)	1.000 (F)
Heart disease	1 (2.7)	0 (0.0)	1.000 (F)
Immune deficiency	0 (0.0)	0 (0.0)	1.000 (F)
Asthma	2 (5.4)	1 (3.2)	1.000 (F)
Other chronic lung diseases	0 (0.0)	0 (0.0)	1.000 (F)
Chronic liver disease	0 (0.0)	0 (0.0)	1.000 (F)
Chronic kidney disease	0 (0.0)	0 (0.0)	1.000 (F)
Hematologic disease\disorder	0 (0.0)	0 (0.0)	1.000 (F)
Chronic neurological impairment\disease	1 (2.7)	1 (2.7)	1.000 (F)
Smoking			
Current	0 (0.0)	0 (0.0)	1.000 (F)
Previous	10 (27.0)	8 (25.8)	0.910

Data presented as n (%); continuous data, mean ± SD; ^†^The body-mass index is the weight in kilograms divided by the square of the height in meters. (F): Fisher’s exact test.

*During COVID19 infection; MoCA, Montreal Cognitive Assessment.

**Figure 1:**
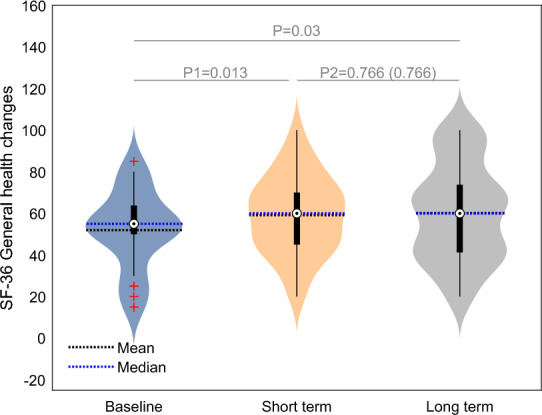
SF-36 General health score changes. Data are presented in a violin plot. Paired t-tests: P1: short term post HBOT compared with base line; P2: long term compared to short term post HBOT; SF-36: Quality of Life questionnaire.

In the context of quality of life assessed by the SF-36 questionnaire, the initial, short -term, evaluation post HBOT revealed substantial and statistically significant enhancements across most domains, except physical functioning as denoted in Table 2. These improvements were characterized by moderate to large effect sizes, indicative of notable clinical impact. Upon protracted assessment, there was an improvement in the physical functioning domain (10.32 ± 21.62, p2 = 0.014), which did not reach statistical significance following correction for multiple comparisons (p2 corrected = 0.111). Other domains did not exhibit statistically significant score ameliorations, as outlined in Table 2. Refer to Fig. 1 for the general health score. Notably, the long-term effect size pertinent to physical functioning exhibited a moderate magnitude, further enhancing the comparatively mild effect size observed 1–3 weeks post HBOT (ES1 = 0.59 vs ES2 = 0.16). A large effect size in both the physical limitations and pain domains were noted at the long term evaluation, compared to the moderate one post HBOT (ES1 = 1.08 vs ES2 = 10.73 and ES1 = 0.86 vs ES2 = 0.62, respectively, Table 2).

**Table 2:** Questionnaires results analysis.

	Baseline	PostHBOT	Long-term	2-month change	Long-term change	P1	P2	ANOVA		ES short term	ES long term
								F (DF)	Sig		
SF36											
Physical functioning	58.39 ± 25.22	61.78 ± 28.01	72.09 ± 22.39	3.38 ± 20.80	10.32 ± 21.62	0.379 (0.379)	0.014 (0.111)	6.45 (2)	**0.002 (0.005)**	0.16	0.59
Physical limitations	16.93 ± 23.23	45.16 ± 37.25	58.87 ± 39.42	28.22 ± 37.96	13.71 ± 35.83	** < 0.001 (0.002**)	0.044 (0.312)	19.63 (2)	** < 0.001**	0.73	1.08
Emotional limitations	32.25 ± 34.37	56.99 ± 38.04	61.29 ± 44.88	24.73 ± 43.96	4.30 ± 42.11	**0.004 (0.013)**	0.580 (1)	7.66 (2)	**0.001 (0.003)**	0.55	0.63
Energy	27.10 ± 17.68	43.87 ± 25.33	46.45 ± 23.83	16.77 ± 25.32	2.58 ± 21.51	**0.001 (0.005)**	0.516 (1)	11.55 (2)	** < 0.001**	0.65	0.77
Emotional wellbeing	49.29 ± 19.10	62.45 ± 21.66	63.61 ± 21.32	13.16 ± 18.41	1.16 ± 14.97	** < 0.001 (0.003)**	0.674 (1)	11.41 (2)	** < 0.001**	0.7	0.68
Social function	44.76 ± 23.06	64.52 ± 25.62	70.16 ± 27.44	19.76 ± 23.71	5.64 ± 20.29	** < 0.001**	0.138 (0.828)	18.46 (2)	** < 0.001**	0.82	0.91
Pain	37.42 ± 34.03	55.97 ± 31.23	60.56 ± 28.97	18.55 ± 29.33	4.59 ± 22.57	**0.002 (0.006)**	0.273 (1)	13.06 (2)	** < 0.001**	0.62	0.86
General health	51.93 ± 18.43	59.19 ± 18.75	60.16 ± 22..23	7.25 ± 15.18	0.97 ± 17.66	**0.013 (0.027)**	0.766 (0.766)	3.88 (2)	**0.025 (0.025)**	0.47	0.41
PSQI											
Global PSQI	10.97 ± 4.00	8.45 ± 4.06	8.22 ± 4.54	− 2.52 ± 3.11	− 0.22 ± 2.47	** < 0.001**	0.620 (1)	17.40 (2)	** < 0.001**	0.79	0.94
Sleep quality	2.06 ± 0.84	1.64 ± 0.90	1.64 ± 0.93	− 0.42 ± 0.87	0.0 ± 0.57	0.013 (0.052)	1 (1)	5.18 (2)	**0.008 (0.033)**	0.47	0.42
Sleep latency	2.00 ± 1.08	1.45 ± 1.24	1.48 ± 1.16	− 0.55 ± 0.80	0.03 ± 0.82	** < 0.001 (0.005)**	0.831 (1)	8.47 (2)	** < 0.001 (0.003)**	0.68	0.61
Sleep duration	1.61 ± 1.10	1.45 ± 0.91	1.35 ± 0.97	− 0.16 ± 0.99	− 0.09 ± 0.53	0.377 (0.377)	0.325 (1)	1.56 (2)	0.219 (0.219)	0.16	0.3
Sleep efficiency	0.58 ± 0.91	0.42 ± 0.83	0.42 ± 0.64	− 0.16 ± 0.51	0.0 ± 0.67	0.096 (0.288)	1 (1)	1.59 (2)	0.212 (0.423)	0.31	0.31
Sleep disturbances	1.87 ± 0.61	1.45 ± 0.50	1.48 ± 0.66	− 0.42 ± 0.66	0.03 ± 0.54	**0.002 (0.009)**	0.744 (1)	8.47 (2)	** < 0.001 (0.004)**	0.62	0.58
Sleep mediation	0.81 ± 1.23	0.48 ± 1.04	0.42 ± 0.83	− 0.32 ± 1.15	− 0.06 ± 0.80	0.134 (0.268)	0.662 (1)	2.67 (2)	0.078 (0.233)	0.28	0.39
Daytime dysfunction	2.03 ± 0.74	1.55 ± 0.84	1.42 ± 0.83	− 0.48 ± 0.91	− 0.13 ± 0.91	**0.007 (0.033)**	0.442 (1)	7.61 (2)	**0.001 (0.005)**	0.52	0.67
BSI-18											
Total	25.67 ± 12.01	16.74 ± 12.55	15.29 ± 12.15	− 8.93 ± 10.74	− 1.45 ± 7.97	** < 0.001**	0.32 (0.98)	18.48 (2)	** < 0.001**	0.81	0.9
Somatization	9.61 ± 5.69	6.55 ± 5.81	5.93 ± 4.78	− 3.06 ± 3.41	− 0.61 ± 3.69	** < 0.001**	0.37 (0.74)	14.71 (2)	** < 0.001**	0.88	0.77
Depression	7.51 ± 5.72	4.16 ± 4.66	4.19 ± 4.42	− 3.35 ± 5.70	0.03 ± 3.68	**0.003 (0.006)**	0.96 (0.96)	9.28 (2)	** < 0.001**	0.58	0.64
Anxiety	8.55 ± 4.36	6.03 ± 4.60	5.16 ± 4.60	− 2.51 ± 4.80	− 0.87 ± 3.17	**0.007 (0.007)**	0.14 (0.57)	10.92 (2)	** < 0.001**	0.51	0.79
BPI											
Pain severity	4.09 ± 2.40	3.11 ± 2.58	3.08 ± 2.24	− 0.97 ± 1.39	− 0.03 ± 1.26	** < 0.001**	0.89	9.39 (2)	** < 0.001**	0.69	0.6
Pain interference	4.85 ± 2.80	2.89 ± 2.84	2.58 ± 2.30	− 1.96 ± 2.33	− 0.31 ± 2.06	** < 0.001**	0.42	18.01 (2)	** < 0.001**	0.83	0.95

Data are presented as mean ± SD; Bold, significant after Bonferroni correction.

ANOVA: repeated measures including baseline, short term and long term measurements.

Paired t-tests: P1: short term post HBOT compared with base line, P2: long term compared to short term post HBOT.

Cohen's d effect size: ES1: short term compared to baseline, ES2: long term compared to short term, ES3: long term compared to baseline.

SF-36: Quality of Life; PSQI: The Pittsburgh Sleep Quality Index; BSI-18: Brief Symptoms Inventory; BPI: Brief Pain Inventory.

Transitioning to the sleep evaluation conducted by the PSQI questionnaire, the short-term evaluation following HBOT underscored notable improvements in various facets, including the global score, sleep quality, sleep latency, sleep disturbances, and daytime dysfunction (p1 < 0.001). These improvements were accompanied by effect sizes of moderate magnitude (ES1 = 0.47–0.79, Table 2).

**Figure 2:**
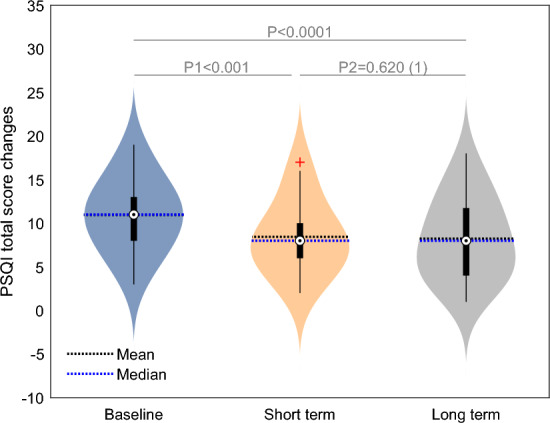
PSQI total score changes. Data are presented in a violin plot. Paired t-tests: P1; short term post HBOT compared with base line; P2: long term compared to short term post HBOT; PSQI: The Pittsburgh Sleep Quality Index.

The improvements at the end of the HBOT sessions, in those 5 sleep domains, were persistent (p2 = 1, Fig. 2). The global PSQI short term medium effect size improved to a large effect size (ES2 = 0.94 vs ES1 = 0.79). All other improved domains moderate effect sizes persisted in the long-term follow-up. Time from the last HBOT session was not a significant covariate for all domains.

**Figure 3:**
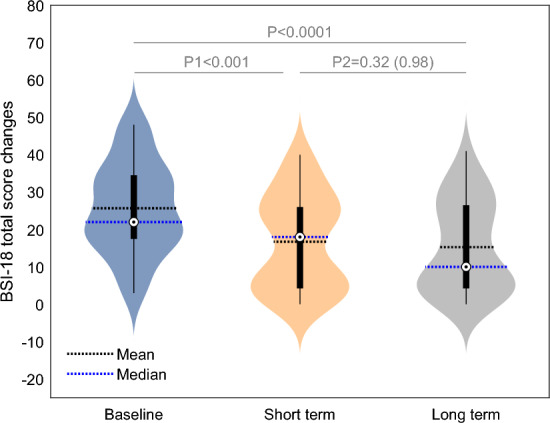
BSI-18 total score changes. Data are presented in a violin plot. Paired t-tests: P1: short term post HBOT compared with base line; P2: long term compared to short term post HBOT; BSI-18: Brief Symptoms Inventory.

In the context of neuropsychiatric symptoms as evaluated by the BSI-18 short-term evaluation subsequent to HBOT, a noteworthy increment was observed in the total score ((− 8.93 ± 10.74, p1 < 0.001) accompanied by a large effect size of 0.81 (Fig. 3). This augmentation extended to specific domains encompassing somatization, depression, and anxiety, each displaying statistically significant improvements (p1 < 0.01) coupled with effect sizes of moderate magnitude (0.88, 0.58, and 0.51, respectively). The beneficial effect of HBOT was persistent, without significant different for the effect achieved at the end of the HBOT in both total and sub-domain scores (p2 > 0.5). It is pertinent to note that the long-term effect sizes mirrored those observed during the short-term evaluation.

In addition, both pain severity (− 0.97 ± 1.39, p1 < 0.001, ES1 = 0.69) and pain interference (− 1.96 ± 2.33, p1 < 0.001, ES1 = 0.83) exhibited significant changes during the short-term assessment post HBOT. In the long-term evaluation, there was non-significant further improvement within these domains (p2 = 0.89 and 0.42). The effect sizes for pain severity (ES2 = 0.6 vs ES1 = 0.69) and pain interference (ES2 = 0.95 vs ES1 = 0.83) remained consistently moderate and large respectively, reinforcing their stability over time.

Time from the last HBOT session was not a significant covariate for all domains in all questionnaires (Table S1).

## Discussion

In this long-term longitudinal follow-up of the active treatment group in our original randomized controlled trial, we found HBOT effects on quality of life, emotional well-being, sleep quality and neuropsychological symptoms in long COVID patients were maintained even more than 1 year (486 ± 73) after the last HBOT session. In all quality of life domains (SF-36), emotional domains (BSI-18), sleep quality (PSQI) domains pain severity and interference, long term scores were not statistically different from the beneficial effects evaluated 1–3 weeks after HBOT. The persisted improvements, more than a year after the last HBOT session, further support the growing knowledge that the new protocols of HBOT induced repairment and neuroplasticity at the biological level and those the clinical effect is not transient.

A recent meta-analysis has showed more than 57% of long covid syndrome patients continue to suffer from symptoms more than 12 months after infection. These main symptoms include frailty, physical limitations, fatigue, and cognitive deficits^19^. The current long term evaluation confirms that the new protocols of HBOT has an effective lasting effect on long covid patients more than 1 year after their treatment.

Long COVID has been linked to persistent psychiatric symptoms, such as depression, anxiety, and somatization^20^. Benedetti et al., identified alterations in brain microstructure, specifically in the superior and posterior corona radiata, superior longitudinal fasciculus, and cingulum, using MRI Diffusion Tensor Imaging (DTI) measures^21^. Additionally, COVID can induce significant perfusion changes in both insula, hippocampus, putamen, prefrontal and cingulate cortex^22–24^, areas associated with pain severity and pathological pain interference. In brain functional and structural connectivity analysis of our original study we have found that HBOT improved disruptions in white matter tracts and alters the functional connectivity organization of neural pathways attributed to cognitive and emotional recovery in post-COVID-19 patients^6^. This correlation between improvements in psychiatric symptoms and MRI-detected changes underscores the biological underpinnings of this condition and the therapeutic impact of HBOT. The finding that the clinical results were preserved after one year, reenforcing the previous findings that these are permanent changes driven by constant microstructural changes, i.e. brain injury recovery.

The pathogenesis of long COVID within the central nervous system involves a multitude of mechanisms^4^. One potential factor is the disruption of the blood–brain barrier (BBB) induced by the cytokine storm, leading to neuroinflammation and neuronal injury. Another possible contributor is the direct neurotropism of SARS-CoV-2^25^. An exaggerated inflammatory response, with elevated levels of TGF-β, has been proposed as a mechanism for the development of neuropsychiatric and other neurological disorders in COVID-19^26^.

Furthermore, the neurological involvement in COVID-19 might be associated with the development of demyelination disorders, as previous coronavirus infections have been linked to neurodegeneration and demyelination^27^. Notably, the high expression of Angiotensin converting enzyme 2 (ACE2) in specific brain regions, such as the substantia nigra and the limbic system, could increase the interaction between SARS-CoV-2 and neurons, potentially leading to neurological complications^28,29^. The dysfunction of GABA-ergic neurons, due to inflammation, and high circulating ACE2 blocking direct activation of pre-sympathetic neurons, have been linked with chronic fatigue and dysexecutive syndrome long covid^30,31^. Lastly, hypervisocity-hypoperfusion syndrome associated with thrombotic events resulting in ischemic incidents, hypoxia, mitochondrial dysfunction,and metabolic dysfunction have also been suggested^32–35^.

**Figure 4:**
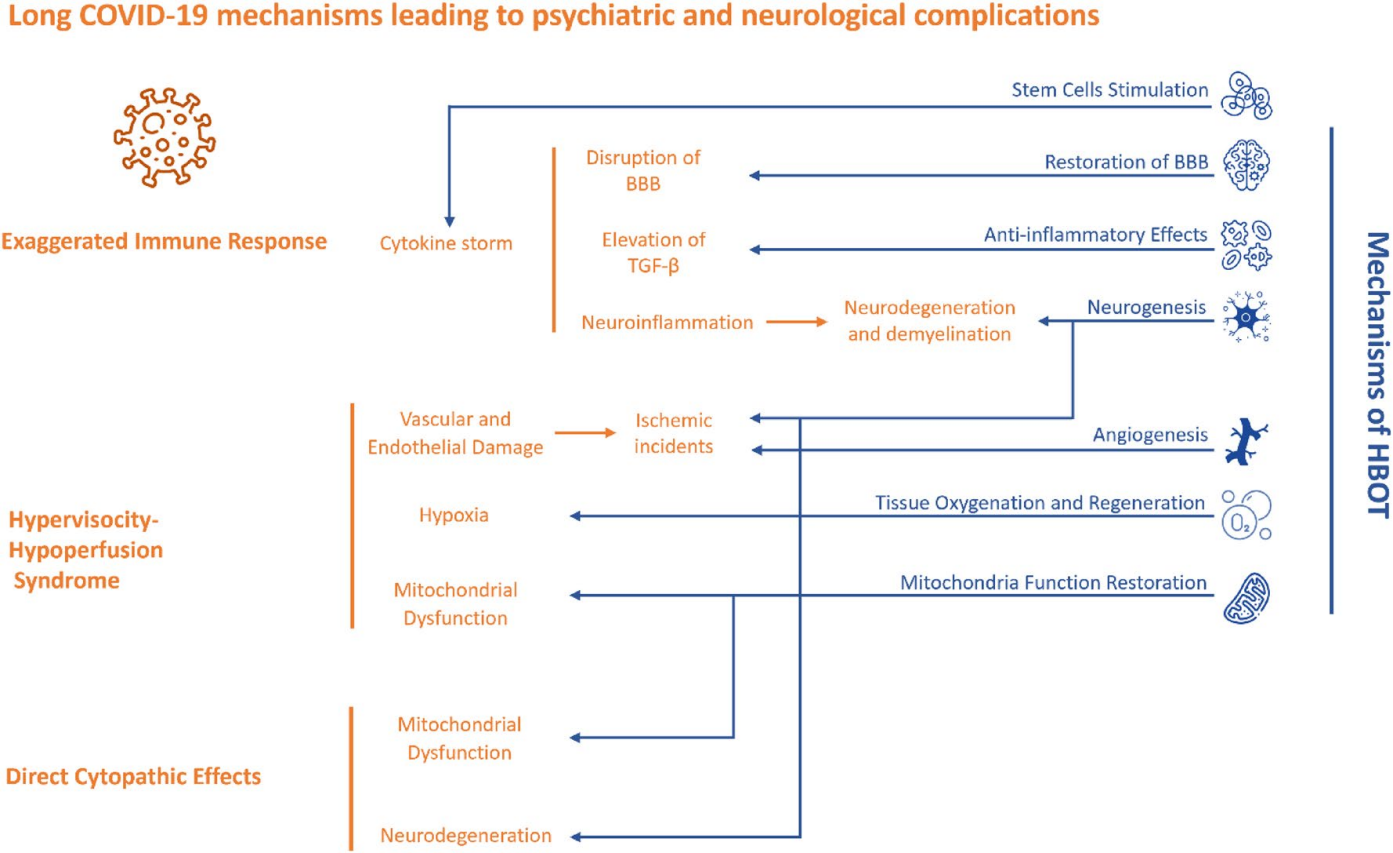
HBOT mechanisms in Long Covid-19 pathophysiology.

These various pathways collectively culminate in dysfunctional brain tissue or chronic brain injury. New HBOT protocols have been recently shown to facilitate neuroplasticity and enhance brain injury recovery, even when administered months or years after the initial injury^12^. These protocols, including the one implemented in our original and present study, leverage the "Hyperoxic-Hypoxic paradox" (HHP). This paradox involves the repeated fluctuations in pressure and oxygen concentrations, which, in turn, trigger the expression of genes and activate metabolic pathways vital for regeneration. Remarkably, this is achieved without exposing the brain to perilous hypoxic conditions^11,13,36^. At the subcellular level, HBOT restores mitochondria function (in both neurons and glia cells) and metabolism^37^, attenuated by long COVID. By delivering high oxygen concentrations, HBOT can enhance oxygen delivery to tissues, reversing the local hypoxia and aiding in recovery of injured tissue^38^. At the cellular level, HBOT anti-inflammatory effects modulates the release of cytokines and inflammation associated with long COVID. By stimulating vasculogenic stem cells, HBOT induces angiogenesis^39^, addressing the vascular damage or thrombosis caused by long COVID. Lastly, HBOT has the ability to facilitate neurogenesis within compromised brain tissue^12–15,17^. It is plausible to suggest that the combination of these effects could serve as the underlying mechanisms contributing to the observed permanent clinical improvements associated with HBOT, as corroborated by the results of our present study (Fig. 4).

Numerous clinical studies have explored the impact of HBOT on neurodegenerative diseases^40^, as well as on other neurological conditions such as stroke and TBI, which may contribute to the development of neurodegenerative diseases like Alzheimer's disease (AD)^40^. Evidence indicates that HBOT induced neuroplasticity with significant improvements in motor and cognitive function among stroke survivors^17,41,42^. HBOT treatment has demonstrated enhancements in both cognitive function and quality of life for chronic TBI patients^41^. Considering that age is a major risk factor for several neurodegenerative diseases, it is crucial to examine the effects of HBOT on the neurobiology of aging. HBOT has shown efficacy in ameliorating age-related cognitive deficits in healthy elderly subjects^18^. Moreover, HBOT exhibits a high safety profile in elderly adults, with barotrauma and visual changes being the primary side effects. Less than a handful of studies have evaluated the long-term effects of HBOT in any neurological indication and brain injury in specifics. Weaver et al. reported improved questionnaire based scores in traumatic brain injury patients post HBOT and 6 months later, while improvements regressed 12 months after HBOT^43^. However, this study did not utilize one of the newer HHP based protocols. Our group has recently reported the long-term effects of such a protocol on military veterans suffering from post-traumatic stress disorder (PTSD) who were treated with HBOT. Similarly, two years post the last HBOT session, the clinical effects were persistent and not attenuated^44^. As mentioned above, these protocols targets injured tissue recovery and true neuroplasticity, which will enable long term clinical effects.

The study has several limitations. First, the sample size was relatively small with 31 patients in total. Second, the primary endpoint in the original study, cognitive function, as well as brain imaging were not evaluated in the current longitudinal evaluation. Third, in the original RCT, patients who received sham intervention were not evaluated long term. Since the original sham group, after completing the study protocol, were offered to be treated with HBOT, and most of them received it (27/39, 69%) they could not serve as a proper control group for the current study.

In conclusion, HBOT can improve the quality of life, quality of sleep, psychiatric and pain symptoms of patients suffering from long COVID. The clinical improvements gained by HBOT are persistent even 1 year after the last HBOT session.

## Methods

### Study design

Longitudinal long-term follow-up of a prospective, randomized controlled trial which included men or women 18 years of age or older with reported post COVID-19 cognitive symptoms that affect quality of life and persist more than 3 months following a confirmed symptomatic SARS-CoV-2 infection and treated with 40 daily HBOT sessions^5^. Patients were excluded if they did not complete either short or long term evaluations. All included patients participated in the original RCT study, registered with clinicaltrials.gov NCT04647656, and approved for long term evaluation by the Shamir medical center institutional review board (approval number 212-22, registered 15/09/2022). All methods were performed in accordance with the international standards based on the declaration of Helsinki.

### Intervention

The protocol comprised of 40 daily sessions, five sessions per week within a 2-month period. The HBOT protocol included breathing 100% oxygen by mask at 2ATA for 90 min with 5-min air breaks every 20 min. Compression/decompression rates were 1.0 m/min.

### Procedure

Patients filled out outcomes questionnaires at baseline, 1–3 weeks after the last HBOT session (short-term evaluation) and one year after their last HBOT (long-term evaluation). One year after their last HBOT session, participants were contacted by phone followed by an electronic mail providing a link to questionnaires. Questionnaires were scored automatically. Submitting the electronic questionnaires was considered as consent, as a written consent was waived by the IRB.

### Study population

Out of 91 eligible patients, 79 were randomized to either HBOT or control/SHAM in the original study. Out of the 40 patients allocated to the HBOT arm, 37 patients completed the intervention and performed the short term evaluation. Of those, six declined their participation in long term evaluation. Accordingly, a total of 31 patients received HBOT, had both short term and long-term post treatment evaluations and were included in the current study analysis. Participants were recruited for the current study after an average of 486 ± 73 days after completion of the last HBOT session.

**Figure 5:**
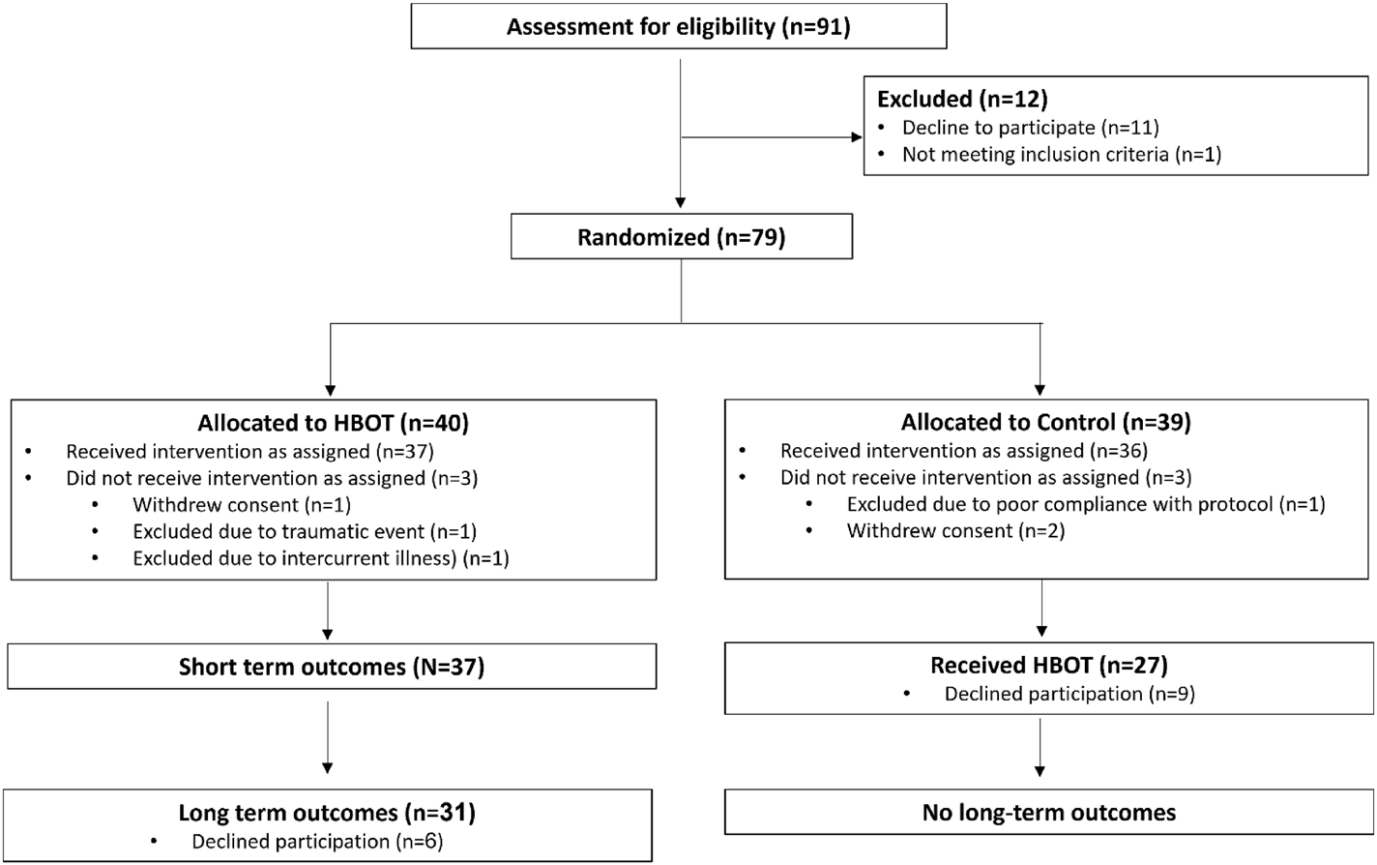
Patients’ flowchart.

The patients’ flowchart is presented in Fig. 5.

### End points

#### Quality of life: Short Form Survey (SF-36)

The short version of the SF-36 questionnaire was employed to evaluate the quality of life of the patients. This questionnaire encompasses the following domains: physical function, role physical, general health, vitality, bodily pain, mental health, role emotional, and social function. The internal consistency of the questionnaire, as measured by the Cronbach's alpha coefficient, was calculated to be 0.94, indicating high reliability^45^. Additionally, the inter-rater reliability, as assessed by the intraclass correlation coefficient (ICC), yielded an high value of 0.98^46^. The overall scores on this index range from 0 to 100, where higher scores correspond to higher quality of life.

#### Neuropsychiatric symptoms: Brief Symptom Inventory (BSI-18)

The BSI-18 questionnaire comprises 18 items, organized into three distinct symptom scales: somatization (6 items), depression (6 items), and anxiety (6 items)^47^. Respondents are asked to rate the severity of each symptom based on a consistent 0–4 scale, reflecting their experiences over the past 7 days. Summing up these individual responses provides a global severity index. The overall scores on this index range from 0 to 72, where higher scores correspond to more pronounced symptom severity.

#### Quality of sleep: Pittsburgh Sleep Quality Index (PSQI)

The PSQI is a widely utilized self-administered assessment tool aimed at measuring sleep quality within clinical populations^48^. Comprising 24 questions, the scale inquires about experiences over the preceding month, assigning ratings on a scale of 0 to 3 for 20 items, while the remaining 4 items are open-ended. Of these, 19 self-reported queries are then utilized to derive scores, categorizing the PSQI into seven components: subjective sleep quality, sleep latency, sleep duration, habitual sleep efficiency, sleep disturbances, use of sleep medications, and daytime disturbance. Cumulatively, the summation of these component scores generates the total score, which spans from 0 to 21. Higher scores correspond to diminished sleep quality. Cutoff scores of 5 and 8 respectively denote "poor" sleepers^48^.

#### Pain: Brief Pain Inventory (BPI)

The BPI, a well-validated questionnaire, serves as an adept tool for evaluating patients' personal experiences of pain^49^. The initial two queries pertain to gauging pain severity on a scale ranging from 0 (denoting the absence of pain) to 10 (representing the highest imaginable pain). The subsequent inquiry pertains to the degree of interference pain has had on the patients' daily lives over the preceding 24 h. Respondents are prompted to provide a score on an interference scale, encompassing values from 0 (indicating no interference) to 10 (indicating significant interference)^50^.

### Statistical analysis

Continuous data were expressed as means ± standard deviations (SD). Two-tailed independent t-tests were performed to compare variables between groups, when a normality assumption was held according to a Kolmogorov–Smirnov test. Effect sizes were evaluated using Cohen's d method. Categorical data were expressed in numbers and percentages, and compared by chi-square/Fisher’s exact tests. To evaluate HBOT’s effect, a repeated measures ANOVA model including baseline, short term and long-term evaluations. Time from the last HBOT session to long term evaluation was evaluated separately as a possible covariate as an in-between factor in the repeated measures ANOVA model. Post-hoc two t-tailed dependent t-tests were used to compare changes between the time points. Multiple comparisons correction was performed Bonferroni. A value of p < 0.05 was considered significant. Sample size was determined by original study. Data analysis was performed using Matlab R2021b (Mathworks, Natick, MA) Statistics Toolbox.

### Supplementary Information

Supplementary Table S1.

## Data Availability

The datasets analyzed during the current study are available from the corresponding author on reasonable request.
