# Supplementary material for: Long term outcomes of hyperbaric oxygen therapy in post covid condition: longitudinal follow-up of a randomized controlled trial
Source: Sci Rep. 2024 Feb 15;14:3604. doi: 10.1038/s41598-024-53091-3 (PMC10869702; doi:10.1038/s41598-024-53091-3)
Supplement: Supplementary file 1 — Supplementary Table S1. [file 41598_2024_53091_MOESM1_ESM.docx]

**Appendix**

Table-S1:

ANOVA with the time from the last HBOT session as a covariate.

|  | F (DF) | pvalue |
| --- | --- | --- |
| **SF36** |  |  |
| Physical functioning | 0.159 (2) | 0.853 (0.853) |
| Physical limitations | 0.613 (2) | 0.545 (1) |
| Emotional limitations | 2.946 (2) | 0.06 (0.483) |
| Energy | 1.905 (2) | 0.158 (0.948) |
| Emotional wellbeing | 2.116 (2) | 0.129 (0.908) |
| Social funtion | 1.013 (2) | 0.369 (1) |
| Pain | 0.546 (2) | 0.582 (1) |
| General health | 0.248 (2) | 0.78 (1) |
|  |  |  |
|  |  |  |
| **PSQI** |  |  |
| Global_PSQI | 0.817 (2) | 0.447 (1) |
| Sleep quality | 1.334 (2) | 0.271 (1) |
| Sleep latency | 0.452 (2) | 0.638 (0.638) |
| Sleep duration | 0.649 (2) | 0.526 (1) |
| Sleep efficency | 1.421 (2) | 0.249 (1) |
| Sleep disturbances | 1.488 (2) | 0.234 (1) |
| Sleep mediation | 2.76 (2) | 0.07 (0.572) |
| Daytime dysfunction | 1.851 (2) | 0.166 (1) |
|  |  |  |
|  |  |  |
| **BSI-18** |  |  |
| Total | 1.096 (2) | 0.341 (1) |
| Somatization | 0.847 (2) | 0.434 (0.434) |
| Depression | 2.018 (2) | 0.142 (0.568) |
| Anxiety | 0.973 (2) | 0.384 (0.768) |
|  |  |  |
| **BPI** |  |  |
| Pain severity | 0.366 (2) | 0.695 |
| Pain interference | 0.666 (2) | 0.517 |
